# On Oxygen Administration

**Published:** 1893-12-16

**Authors:** Benjamin Ward Richardson


### On Oxygen Administration.

By Sir Benjamin Ward Richardson, M.D., F.R.S.

I promised in my last essay to try and afford some reasons why, in the treatment of disease by oxygen—in phthisis pulmonalis especially—different results occur in cases that seem entirely alike. The most ready impression is that the differences, which seem always to have been acknowledged, are due to errors of diagnosis. In the course of treatment of large numbers of phthisical patients there are invariably a certain exceptional number who recover, and the rule has been to consider that these, after all, were not examples of the actual disease. This has been the rule of thought of all honest men, while quacks, having a specific remedy of their own, have often flourished on such exceptions.

I believe there is some considerable truth in the candid view that has thus been taken, yet it is not the whole truth, and when we employ oxygen as a remedy, not only should we carefully exclude every recovery where there is any doubt in diagnosis, but we should look upon oxygen, in its effects upon human life, as subject to variability according to the conditions in which it is applied, and to the physical condition of the gas itself at the time it is applied.

After many years of patient research in regard to oxygen gas, in experience as well as in experiment, I find more reasons for want of uniformity of action than from serious errors in diagnosis. In the first place, the gas varies considerably in regard to its active state, and thereby, according to its combining power. The gas that has been long kept compressed in an iron bottle is not the same as that which is freshly made and received, well washed, into a fresh receiver. I pointed out this fact to the late Dr. Edward Smith, and he in his researches on the exhalation of carbonic acid by the breath after the subjection of the body to special agencies—foods, drinks, and gases—told me he was quite unprepared to meet the changes brought about by the simple modification above suggested. Freshly made oxygen quickens the pulse, produces a glow over the cutaneous surface, causes slight exhilaration, and increases respiration. Oxygen held long under compression in the iron tube becomes negative in these respects, a result which unfortunately tells against the most convenient mode we have at present at command for administering the gas.

Heat and cold make the most distinct differences in regard to the action of oxygen. The inhalation of the gas reduced in temperature is attended with results of a marked order when the gas is inhaled long enough to produce observed effects. I found, by direct experiments, that when warm blooded animals were immersed in the gas reduced in temperature to freezing point they became torpid, and that the temperature of their bodies fell below the normal. Once, indeed, I carried this reduction of temperature so far that the cold gas became an anæsthetic. In this state, and in states where it was not reduced to any such negation as this, the value of the gas as an excitant was lost. On the other hand, the gas raised to considerable heat has just the opposite effect. Above 80 deg. Fahrenheit oxygen causes the body inhaling it to increase in vital activity. The temperature of the body may remain nearly the same or be only a little elevated, because the free excretion that follows equalises the animal fire; but the symptoms are of another character altogether. The body is relaxed in tone, the venous blood is rendered arterial in colour, and in lower animals the desire for food and water is remarkably increased, while the body generally is restless and not inclined to torpor.

Again, the action of oxygen is modified in another and extreme degree by the influence of electricity, and of electrical conditions. We all know now, that under the electric discharge oxygen becomes condensed, and the substance called ozone formed. The inhalation of electrified oxygen, even if it be in comparatively diluted form, is followed by symptoms absolutely peculiar to itself. I made this a matter of careful experiment, on myself and on lower animals. The gas in this state acts as a local irritant, it produces pricking and soreness of the nostrils and eyelids, succeeded by headache more or less violent, and if long continued, by difficult breathing, congestion of the lungs, congestion of the right side of the heart, and—in lower animals—coagulation of the blood, and death.

I have briefly sketched out so much of a physiological kind relating to oxygen as to show that, without a degree of precision we have not yet arrived at, uncertain effects must attend its therapeutical employment. In its negative state it may be less effective than common air, and less easily absorbed into the blood; but it might then be used with advantage in acute febrile diseases. In conditions of high temperature it produces free diaphoresis and active elimination, and might prove of immense service in toxic dangers. I had once a good illustration of its value in this last respect. I was taken to see a patient suffering from tetanus, who was being kept under pure oxygen inhaled from a Barth's reservoir. There had been great relief from the inhalati on; the tetanic spasms were subdued, and recovery, which did ultimately take place, was in prospect. At the period of my visit there was no tetanic paroxysm, and the muscles over the whole of the body were much relaxed—a sign which was in every sense favourable. But the patient was in the most profuse perspirations, and had been so from the time—forty-eight hours—during which the oxygen treatment had continued. These symptoms, induced under the oxygen, were, however, not caused by the oxygen alone; they were aided by the temperature. The temperature of the day was from 75 deg. to 80 deg. Fahrenheit. I explained at the time that the good signs were due to this combined action of heat and oxygen, the facts agreeing in the most positive way with experimental data.

In another instance when oxygen, commonly so called, was being administered, it had to be suspended owing to the circumstance that it brought on headache and coryza. The reason was plain enough. Some charge of ozone had been formed, and to that the unpleasant symptoms were easily referable. The gas had been made in the ordinary way, and had twice been used from the same source without any evil; but now, as examination of it showed, it gave reactions of ozone. Here some atmospherical disturbance had interfered, and had charged it with a new quality.

The therapeutist who resorts to oxygen as a remedy has much to learn before he can expect any approach to uniform results. As it is at present at his command, the gas plays him three different parts at least, and will do so until, by better methods, it is brought to be a medical weapon of precision; probably then the most important of all we have in our medical armamentaria.

